# Salivary Markers for Periodontal and General Diseases

**DOI:** 10.1155/2016/9179632

**Published:** 2016-04-06

**Authors:** Stepan Podzimek, Lucie Vondrackova, Jana Duskova, Tatjana Janatova, Zdenek Broukal

**Affiliations:** School of Dental Medicine, First Faculty of Medicine and General University Hospital, Charles University, Karlovo Namesti 32 and Katerinska 32, 121 11 Prague, Czech Republic

## Abstract

The determination of biomarkers in saliva is becoming an important part of laboratory diagnostics and the prediction of not only periodontal, but also other tissue and organ diseases. Biomarkers in saliva (e.g., enzymes, protein markers, or oxidative stress markers) can be used for activity determination and for periodontal disease prognosis. Saliva also contains many markers which can predict the risk of certain diseases (e.g., diabetes mellitus, cardiovascular, oncology, endocrinology, and psychiatric diseases). The study of salivary components proteomics clearly shows the relationship of periodontal diseases and diseases of distant systems, organs, or tissues.

## 1. Introduction

Saliva is an accessible biofluid that contains components derived from the mucosal surfaces, gingival crevices, and tooth surfaces of the mouth. Saliva also contains microorganisms that colonize the mouth and other exogenous substances and so can potentially provide an insight into the relationship of the host with the environment [1].

Research on the composition of the saliva and the presence of periodontal and other disease markers became intensive again thanks to the development of laboratory nanotechnologies that pushed detection limits of various metabolites, signal molecules, hormones, and other substances by several orders of magnitude. In addition to molecular methods, analytical chips are also available that may, by merely changing the detection plates, detect a series of various chemical substances.

Most metabolites, cytokines, signal molecules, or hormones move in a certain amount by passive filtration into saliva and their levels in saliva reflect their levels in plasma. Therefore, detection of these molecules in saliva is only a matter of the detection limits of new analytical methods. The potential of saliva as a biomarker fluid has been transformed by the development of highly sensitive proteomic analysis, which has identified the presence of over 2,000 proteins, approximately 25–30% of which are shared with blood [2].

It is also important to mention that an advantage of using saliva, as a diagnostic material, is that it can be obtained for laboratory tests noninvasively and repeatedly [3].

Saliva is a very complex system which includes not only both its own components and sulcular fluid components, microorganisms, and products of inflammation ongoing in periodontium, but also metabolites and signal molecules accompanying remote processes. Some components of saliva can come from multiple sources, such as proteolytic enzymes. These can come not only from polymorphonuclear leukocytes and periodontal microorganisms, but also from the bloodstream. It is similar to the products of inflammation which may also have both local and systemic origin.

Enzymes, specific and nonspecific proteins, antibodies, and other substances are among the potential salivary biomarkers of periodontal and certain distant tissue diseases. And so saliva became the topic of interest among experts in proteomics, research of sequential composition of individual proteins.

A potential limitation in the use of saliva as a diagnostic fluid is oral dryness caused by the failure to produce saliva. Dry mouth is most frequently the side effect of the consumption of many prescribed medications and is more prevalent in older age groups. A high degree of medication-induced dryness, resulting from salivary hypofunction, is associated with anticholinergic muscarinic receptor blockers used to treat, for example, irritable bladders. Antidepressants can also cause salivary hypofunction as a result of the activation of alpha-2-adrenergic receptors in the central nervous system. The most severe forms of long-lasting, irreversible dry mouth are seen in patients with squamous cell carcinoma given external irradiation of the head and neck and in patients with Sjögren's syndrome [1].

In this review, we will only mention some of the biomarkers of periodontal diseases and some distant diseases in saliva to illustrate the direction of this research. This review does not focus on biomarkers in gingival crevicular fluid; such review has been recently published in Periodontology 2000 journal [4, 5].

Three limitations prevent the full benefits of clinical diagnostics from being realized: definitive biomarkers associated with disease; simple and inexpensive methods that are minimally invasive; and an accurate, portable, and easy-to-use diagnostic platform [6].

It is certain that more research effort is necessary to determine the sensitivity and specificity of salivary biomarker detection and to increase the availability of routine detection methods [7, 8].

## 2. Saliva as a Source of Markers for Periodontal Diseases

### 2.1. Enzymatic Activity in Saliva

#### 2.1.1. Aspartate Aminotransferase (AST)

One of the longest studied markers of inflammation is aspartate aminotransferase. It belongs among transaminases which have been investigated for many years in clinical biochemistry. The enzyme catalyzes the transamination of glutamic acid to oxaloacetic and aspartic acids. During inflammation, AST tissue level rises; it gets into the blood plasma and also by diffusion through salivary glands into saliva. During periodontal inflammation, it also passes into sulcular fluid and then into saliva. AST levels are significantly and positively correlated with the intensity and extent of periodontal inflammation [9].

Periodontal disease progression, as defined by pocket depth, gingival bleeding, and suppuration, is linked with increased levels of salivary aspartate aminotransferase [10]. Periodontal scaling treatment causes a marked decrease in aspartate aminotransferase levels [11].

In periodontology chairside detection kits are already available, known under the names PerioGard, Hawe Perimonitor, or Pocketwatch. For the detection of AST in plasma or saliva, Reflotron multifunction system is now available.

#### 2.1.2. Proteinases

Neutrophils and other phagocytic cells release specific proteinases, dipeptidyl peptidases and aminopeptidases. Their levels in saliva correlate with the intensity and extent of inflammation in periodontal tissues [12, 13].

#### 2.1.3. Lactoferrin

In addition to the increased levels of proteinases, the level of lactoferrin in saliva during periodontal inflammation also increases. It is known that, during periodontitis, lactoferrin saturation with iron decreases; therefore lactoferrin degrades into products that can damage tissue [14, 15].

#### 2.1.4. Metalloproteinases

Many scientific articles focus on metalloproteinases and their relationship to periodontal inflammation because collagen diseases may induce periodontal inflammation. Today more than twenty metalloproteinases isoenzymes and their tissue inhibitor systems are known. In patients with periodontitis, it has been proven that increasing metalloproteinases simultaneously decreases their tissue inhibitors in saliva [16].

Matrix metalloproteinases 8 and 9 are the major matrix metalloproteinases in saliva; in smaller amounts, saliva also contains matrix metalloproteinases 1, 2, 3, 7, 12, 13, 14, 25, and 26 and tissue inhibitors of matrix metalloproteinases 1 and 2 [17].

Increased bleeding upon probing, clinical attachment level, and pocket depth have repeatedly demonstrated significant, positive correlations with elevated levels of salivary matrix metalloproteinase 8 [18, 19].

After periodontal scaling and root planning, significant reductions in salivary matrix metalloproteinase 8 levels have been found, suggesting matrix metalloproteinase 8 potential marker for monitoring periodontal disease activity [20].

Matrix metalloproteinase 9 mediates many functions of the periodontal disease process, including tissue destruction and immune responses. Elevated salivary levels of matrix metalloproteinase 9 were found in clinically diseased conditions and reduced salivary levels were found in clinically stable conditions [21]. Higher levels of matrix metalloproteinase 9 have been detected in the saliva of subjects with periodontitis than in the saliva of controls [22] and, after periodontal therapy, the amount of matrix metalloproteinase 9 decreases [23].

Matrix metalloproteinase 9 appears to be associated with cardiovascular disease, cancer, multiple sclerosis, and neuropsychiatric disorders, such as schizophrenia and bipolar mood disorder [21].

Reviews on matrix metalloproteinases, especially MMP-8, in saliva and other oral cavity fluids for monitoring periodontal diseases have been recently published in Periodontology 2000 journal [24].

#### 2.1.5. Chitinase and Hexosaminidase

Chitinase and hexosaminidase are antibacterial active enzymes derived from phagocytic cells. Their activity in saliva replicates that of periodontal inflammation [25].

### 2.2. Nitric Oxide Radical (NO)

Nitric oxide radical is important for the proper function of neutrophils and macrophages. Its production is controlled by reciprocal countercurrent interaction of NO synthase and arginase with the participation of neopterin. In patients with periodontitis, the arginase level in saliva decreases. It is assumed that this can be a consequence of a decrease in phagocytic activity [26].

### 2.3. Other Markers in Periodontal Diseases

Upregulated expression of a variety of B-cell lymphoma-2-related transcripts in neutrophils from oral rinse samples of patients with chronic periodontitis compared with healthy controls has been demonstrated using the Illumina Human 12WG Expression BeadChip (Illumina, San Diego, CA, USA) [27].

Korte and Kinney [28] have recently published an updated review of salivary biomarkers for periodontal diseases.

## 3. Specific and Nonspecific Proteins Present in Saliva

In saliva, the numbers of proteins, which also reflect the state of inflammation in the periodontium and occasionally even the state of inflammation in distant organs systems, are detectable.

The number of salivary biomarkers is increasing rapidly and many of these markers appear to be unique to saliva. From a list of 1,939 salivary proteins that has been recently published [29, 30] around two-thirds have not been found in blood. This may lead to the discovery of protein “signatures” indicative of oral and systemic diseases [31–33].

### 3.1. C-Reactive Protein (CRP)

C-reactive protein is a long known indicator of inflammatory or rheumatic activity; CRP levels in plasma and saliva correlate. During periodontitis, salivary CRP levels increase and its decrease indicates a successful anti-inflammatory treatment.

High CRP levels in saliva during diffuse periodontitis are a marker of local process risk for the formation or progression of cardiovascular disease with risk coefficient OR 5.6 [34–36].

CRP levels increase subsequently with the severity of the periodontal disease [37].

### 3.2. Fibronectin

During periodontitis, a decreased level of fibronectin in saliva is evident. Fibronectin blocks adhesins of many periodontal microorganisms, reducing their adherence to periodontal tissues [38].

### 3.3. Cystatins

The levels of cystatins, natural inhibitors of phagocytic proteases, decrease in sulcular fluid and saliva during periodontitis, ultimately leading to tissue damage by cathepsins [39].

### 3.4. Neopterin

Neopterin is a cytokine produced by macrophages that participates in the formation of nitric oxide radical which is important for phagocytosis. Its level in saliva increases during periodontal inflammation [26].

### 3.5. Cytokines and Other Protein Markers

Further protein markers of inflammation whose levels correlate with the intensity of periodontal inflammation are detectable in saliva. Examples of these include platelet-activating factor, vascular endothelial growth factor, and hepatocyte growth factor [40–42].

Salivary interleukin-1 beta is elevated in periodontal diseases and its levels are strongly correlated with periodontal disease progression and considered to be a good biomarker for discriminating between active and inactive periodontal sites. Salivary interleukin-1 beta has also been associated with advanced periodontitis [43].

According to recent review, interleukin-1 beta and hepatocyte growth factor are the most robust salivary biomarkers for periodontal disease [44].

Increased salivary levels of interleukin-6 are present in individuals with periodontitis compared with healthy subjects [45]. Salivary interleukin-6 has also been shown to stimulate osteoclast differentiation and bone resorption and is associated with tissue destruction in peri-implant disease [46].

Salivary levels of tumor necrosis factor-alpha indicate the presence of generalized chronic periodontitis and have also demonstrated significant changes after nonsurgical periodontal therapy [20].

A great deal of work has been dedicated to cataloging the salivary proteome and annotating identified saliva proteins [47]. This large database is known as the Saliva Proteome Knowledge Base and is available to the public (http://www.skb.ucla.edu/). This central database is crucial in the identification of combinations of biomarker panels that may provide distinct diagnostic information and thus serves as a valuable resource for identifying deviations within proteins or peptides that align with oral and systemic disease and will aid in the development of saliva-based diagnostic tests [48].

## 4. Oxidative Stress

One of the important molecular pathological processes accompanying inflammation or tumor cell proliferation is the so-called oxidative stress. In the pathologically altered tissue, a number of substances with the character of reactive oxygen species release and their compensation by the present antioxidants are sustained. The equilibrium is disturbed either by increased production of reactive oxygen species, by the lack of antioxidants, or by both mechanisms. This results in damage to the cellular DNA, certain signal proteins and tissue components, inactivation of enzymes, and stimulation of proinflammatory cytokines and lipid peroxidation. It has been proven that oxidative stress significantly succeeded in the destruction of periodontal tissues [49].

One of the markers of oxidative stress is 8-hydroxy-2′-deoxyguanosine (8-OHdG), which is formed by the oxidation of guanine from damaged DNA. Deoxyguanosine levels increase in saliva during periodontitis and decrease with successful anti-inflammatory treatment [50].

During chronic inflammation, not only reactive oxygen species increase in affected tissues, but also a decrease in antioxidants can be demonstrated. This is true even for periodontal tissues [51]. This relationship is supported by results from the study by Takane et al. (2005) which showed significant differences between the levels of 8-OHdG in saliva in patients with aggressive and advanced periodontitis and in individuals with clinically healthy periodontal tissues [52].

Ascorbate and albumin belong to the main tissue antioxidants together with melatonin. Melatonin has nonspecific anti-inflammatory and osteogenic effects, which affects T lymphocytes, and its level in saliva positively correlates with the intensity and extent of periodontal inflammation [53].

Reduced production of interleukin-2 known in patients with type 1 diabetes, together with a reduced level of melatonin, may be one of the molecular pathological risks for the progression of periodontal disease in diabetic patients [54].

Reactive oxygen species also play a role in another molecular pathological process known as lipid peroxidation. Reactive oxygen species attack the unsaturated fatty acids of phospholipid and phosphoprotein membranes, thereby disrupting their integrity. The end products of lipid peroxidation are malondialdehyde and hydroxyalkenals. If the lipid peroxidation is in equilibrium with tissue glutathione and its peroxidase, damage to the membranes does not occur. During periodontitis, elevated levels of the end products of lipid peroxidation and decreased levels of natural antioxidants are found in saliva, indicating failure of equilibrium with the risk of tissue destruction [55].

Reactive oxygen species are implicated in the pathogenesis of many diseases and have been suggested to play a role in the pathogenesis of various oral diseases. For example, the salivary levels of antioxidant enzymes, including superoxide dismutase, catalase, and glutathione peroxidase, were significantly higher in patients with recurrent aphthous stomatitis than in healthy controls [56].

## 5. Saliva as a Source of Biomarkers for Other Fields of Medicine

Studies investigating the use of saliva as a diagnostic fluid have a long history. This noninvasive approach is not limited only to the diagnosis of oral diseases, because many systemic diseases, such as different types of cancers, cardiovascular diseases, immunologic syndromes, and hereditary deficiencies, can also be studied with the aid of salivary diagnostics [57]. Even gynecology and psychiatry are interested in salivary biomarkers monitoring.

Reviews on salivary markers in dental caries [58] and oral mucosal diseases [59] have been recently published in Periodontology 2000 journal.

### 5.1. Diabetes Mellitus

Nonenzymatic glycation of a number of substances occurs during elevated or fluctuating blood glucose levels. The glycated hemoglobin level in blood has long been used as a marker of metabolically poorly controlled diabetes. More recently, it was shown that even in saliva some glycated proteins and fats can be detected, and their levels correlate with the glycated hemoglobin level in plasma [60].

According to some published studies, it appears that levels of sorbitol and fructosamine in saliva correlate with levels of glucose in capillary blood [61].

Increased plasma glycohemoglobin is also accompanied by elevated levels of epidermal growth factor, nitric oxide, and total antioxidant activity of saliva [62].

In patients with type 1 diabetes, increased levels of urea and *α*-amylase have been shown in saliva. According to the study of Lopez et al. (2003), the increase of these markers in saliva precedes the clinical signs of diabetes. These markers may be used in the future for screening incipient diabetes [63].

### 5.2. Cardiovascular Diseases

Wolfram et al. (2005) demonstrated increased levels of isoprostane and oxidized low-density lipoproteins in saliva of individuals with coronary heart disease [64].

Increased attention turns also to endothelin in saliva. Endothelin is a peptide that plays a critical role in cardiovascular regulation. Endothelin receptors mediate vasoconstriction as a response to endothelin-1, the primary isoform of this peptide in the cardiovascular system. It is assumed that endothelin A receptors induce the proliferation of vascular smooth muscle and myocardial hypertrophy, whereas the activations of endothelin B receptors lead to vasodilation via nitric oxide or prostacyclin and have antithrombotic and antiproliferative effects. Pulmonary receptors of endothelin B in addition help to scavenge endothelin-1. Endothelin-1 stimulates the production of other neurohumoral factors and promotes the activity of other neuroendocrine cascades. In people with coronary heart disease or chronic obstructive pulmonary disease, elevated levels of endothelin and its inhibitor are found in saliva [65].

In terms of cardiovascular biomarker research, the feasibility and utility of a programmable bionanochip to identify biomarkers of acute myocardial infarction in saliva were investigated. Interestingly, this nanotechnology was able to identify saliva-based biomarker panels that had significant diagnostic capability when used in conjunction with an electrocardiogram and far exceeded the screening capacity of an electrocardiogram alone [66].

### 5.3. Oncological Screening

In oncological screening, the detection of soluble factor CD44 or telomerase is being considered as their levels in saliva increase with squamous cell carcinoma of the head and neck [67, 68]. High attention is still paid to the possibilities of detecting markers for oncological diseases of the oral cavity [69, 70].

In proteomics research of plasma and saliva protein composition, it was found that, in patients with breast or ovarian cancer, the fractions with molecular weight of 117, 228, and 287 kDa significantly increased in plasma and saliva. It seems that the biomarker of these malignancies could be protein, provisionally named as HER2/neu [71].

CA 125 antigen is present in the plasma of ovarian cancer patients, and its detection is used as a marker for recurrence after oncologic treatment. This marker is also detectable in saliva, and a possible use of CA 125 determination in saliva is considered in the screening of this cancer [72].

Using Affymetrix HG-U133-Plus-2.0 array (Affymetrix, Santa Clara, CA, USA), four salivary mRNA biomarkers (v-Ki-ras2 Kirsten rat sarcoma viral oncogene homolog, methyl-CpG binding domain protein 3-like 2, acrosomal vesicle protein 1, and dolichylphosphate mannosyltransferase polypeptide 1) that can differentiate patients with early-stage resectable pancreatic cancer from noncancer subjects were identified [73]. This is a breakthrough proof-of-concept investigation for noninvasive detection of a systemic cancer.

Gene panels of 4 to 10 genes for oral squamous cell carcinoma were constructed based on significant differences in DNA-methylation patterns between the preoperative and postoperative saliva of patients with oral squamous cell carcinoma and between preoperative saliva from patients with oral squamous cell carcinoma and saliva from healthy controls [74].

Three salivary metabolites (phenylalanine, valine, and lactic acid) could distinguish patients with oral squamous cell carcinoma from healthy controls and could also distinguish patients with oral leukoplakia from healthy controls [75].

A combination of three microbiotas (*Capnocytophaga gingivalis*,* Prevotella melaninogenica,* and* Streptococcus mitis*) in saliva could be used as diagnostic biomarkers of patients with oral squamous cell carcinoma [76].

Three proteins (zinc-a-2-glycoprotein, haptoglobin, and human calprotectin) that had good discriminatory power in lung cancer patients and healthy control subjects were reported by Xiao et al. [77].

### 5.4. Gynecology

Increased levels of estriol in saliva predict the risk of premature birth. There is a range of detection sets for self-diagnostics of premature birth risk on sale in the USA. An example is the Salivary Estriol Immunoassay Kit from Salimetrics LLC.

Dynamics of estradiol levels in saliva is used in assisted reproduction to accurately determine the ovulation [78, 79].

### 5.5. Endocrinology

Salivary cortisol has long been used in endocrinological examination methods as a biomarker for determining hypothalamus-pituitary-adrenal axis activity [80].

Another salivary marker used by endocrinologists is salivary *α*-amylase (sAA) that helps determine sympathetic-adrenal medulla axis activity. The amount of salivary *α*-amylase significantly increases with adrenergic activation, especially with stress [81].

When hyperandrogenemia occurs, for example, in polycystic ovary syndrome, increased levels of dehydroepiandrosterone sulfate (DHEA-S) and androstenedione can be detected in saliva [82].

### 5.6. Psychiatry

Research in psychiatry also has its markers that can be measured in saliva. It is, for example, the ratio of cortisol and dehydroepiandrosterone which increases in depressive conditions and varies with changes in affectivity. The good predictive value of this assay is used for the individual dosing of antidepressants in adolescents [83].

It has been found that 3-methoxy-4-hydroxyphenylglycol (one of the noradrenaline metabolites) level in saliva decreases in depression or in reduced mental activity. Nowadays, its determination is used for research purposes only [84].

Two salivary metabolites (arginine and tyrosine) differed significantly between patients with dementia and healthy subjects [85].

### 5.7. Drug Addiction

During drug addiction control, the saliva, along with urine, becomes an important material for detecting amphetamine, opiates, phencyclidine, marijuana, and cocaine and their metabolites. There are already a number of special detection kits, ToxCup® (Clia Laboratories), Saliva MultiDrug Screen test kit® (Medimpex Inc.), iScreen® (BioCheck), QuickScreen® (Craig Medical Inc.), and so forth.

### 5.8. Virus Diseases

Saliva HIV tests have demonstrated comparable accuracy to the traditional blood test. Oral HIV tests can be a powerful tool for high-risk populations [86].

For HIV viral-load analysis, a frequently used commercial assay is the COBAS Amplicor HIV-1 Monitor Test (Roche Diagnostics, Indianapolis, USA) and an example of a commercial assay based on signal amplification is the VERSANT HIV-1 RNA Assay (Siemens AG, Munich, Germany) [87].

Hepatitis C virus RNA can be consistently detected in saliva from hepatitis C virus-infected individuals using quantitative PCR [88].

Dengue virus specific IgA in saliva was detected soon after infection in dengue-endemic regions [89].

For the detection of human antibodies against measles and mumps in oral fluid several simple platforms for antibody detection in saliva have been published [90].

Currently, only two oral-based commercial tests for infectious disease are FDA approved for use in the USA: the OraQuick oral HIV test and the OraRisk human papillomavirus salivary diagnostic test [87].

## 6. Conclusion

The determination of biomarkers in saliva is becoming an important part of laboratory diagnostics and the prediction of periodontal and other diseases. Biomarkers in saliva may also serve to determine the context of periodontal disease with an altered state of the body or with chronic diseases. Saliva also contains a series of markers, which may predict the risk of some systemic disease development.

It is certain that a lot more research efforts are necessary to determine the sensitivity and specificity of salivary biomarker detection and to increase the availability of routine detection methods.

The UK Biobank holds the world's largest bank of around 85,000 well-characterized saliva samples, each accompanied by blood and urine samples, the results of a battery of physiological tests, a full medical history, and a detailed description of the subject's lifestyle [91]. The evaluation of these samples can be compared with clinical status of project participants in future and may bring a lot of recently unavailable pieces of information about salivary biomarkers.
